# Plasma PCSK9 concentrations during an oral fat load and after short term high-fat, high-fat high-protein and high-fructose diets

**DOI:** 10.1186/1743-7075-10-4

**Published:** 2013-01-08

**Authors:** Bertrand Cariou, Cédric Langhi, Maëlle Le Bras, Murielle Bortolotti, Kim-Anne Lê, Fanny Theytaz, Cédric Le May, Béatrice Guyomarc’h-Delasalle, Yassine Zaïr, Roland Kreis, Chris Boesch, Michel Krempf, Luc Tappy, Philippe Costet

**Affiliations:** 1 INSERM, UMR 1087, F-44000, Nantes, France; 2 Université de Nantes, Faculté de Médecine, Institut du Thorax, F-44000, Nantes, France; 3 CHU de Nantes, Clinique d'Endocrinologie, Institut du Thorax, F-44000, Nantes, France; 4 Department of Physiology, University of Lausanne, Lausanne, Switzerland; 5 Department of Clinical research, MR Spectroscopy and Methodology, University of Bern, Bern, Switzerland; 6 Division of Diabetes, Endocrinology and Metabolism, Lausanne University Hospital, Lausanne, Switzerland; 7 Present address: SUNY Downstate Medical Center, Department of Cell Biology, 450 Clarkson Avenue, 11203, New York, NY, USA

**Keywords:** Nutrition, Dietary intervention, PCSK9, Insulin resistance, Liver steatosis

## Abstract

**Background:**

PCSK9 (Proprotein Convertase Subtilisin Kexin type 9) is a circulating protein that promotes hypercholesterolemia by decreasing hepatic LDL receptor protein. Under non interventional conditions, its expression is driven by sterol response element binding protein 2 (SREBP2) and follows a diurnal rhythm synchronous with cholesterol synthesis. Plasma PCSK9 is associated to LDL-C and to a lesser extent plasma triglycerides and insulin resistance. We aimed to verify the effect on plasma PCSK9 concentrations of dietary interventions that affect these parameters.

**Methods:**

We performed nutritional interventions in young healthy male volunteers and offspring of type 2 diabetic (OffT2D) patients that are more prone to develop insulin resistance, including: i) acute post-prandial hyperlipidemic challenge (n=10), ii) 4 days of high-fat (HF) or high-fat/high-protein (HFHP) (n=10), iii) 7 (HFruc1, n=16) or 6 (HFruc2, n=9) days of hypercaloric high-fructose diets. An acute oral fat load was also performed in two patients bearing the R104C-V114A loss-of-function (LOF) PCSK9 mutation. Plasma PCSK9 concentrations were measured by ELISA. For the HFruc1 study, intrahepatocellular (IHCL) and intramyocellular lipids were measured by ^1^H magnetic resonance spectroscopy. Hepatic and whole-body insulin sensitivity was assessed with a two-step hyperinsulinemic-euglycemic clamp (0.3 and 1.0 mU.kg^-1^.min^-1^).

**Findings:**

HF and HFHP short-term diets, as well as an acute hyperlipidemic oral load, did not significantly change PCSK9 concentrations. In addition, post-prandial plasma triglyceride excursion was not altered in two carriers of PCSK9 LOF mutation compared with non carriers. In contrast, hypercaloric 7-day HFruc1 diet increased plasma PCSK9 concentrations by 28% (p=0.05) in healthy volunteers and by 34% (p=0.001) in OffT2D patients. In another independent study, 6-day HFruc2 diet increased plasma PCSK9 levels by 93% (p<0.0001) in young healthy male volunteers. Spearman’s correlations revealed that plasma PCSK9 concentrations upon 7-day HFruc1 diet were positively associated with plasma triglycerides (r=0.54, p=0.01) and IHCL (r=0.56, p=0.001), and inversely correlated with hepatic (r=0.54, p=0.014) and whole-body (r=−0.59, p=0.0065) insulin sensitivity.

**Conclusions:**

Plasma PCSK9 concentrations vary minimally in response to a short term high-fat diet and they are not accompanied with changes in cholesterolemia upon high-fructose diet. Short-term high-fructose intake increased plasma PCSK9 levels, independent on cholesterol synthesis, suggesting a regulation independent of SREBP-2. Upon this diet, PCSK9 is associated with insulin resistance, hepatic steatosis and plasma triglycerides.

## Background

PCSK9 (Proprotein Convestase Subtilisin Kexin Type 9) is the ninth member of the proprotein convertase (PC) family [1]. Genetic studies have demonstrated that PCSK9 is a major determinant of cholesterol homeostasis [2,3]. Gain-of-function (GOF) *PCSK9* mutations are associated to autosomal dominant hypercholesterolemia and premature atherosclerosis [4]. In contrast, loss-of-function (LOF) *PCSK9* mutations lead to low concentrations of plasma low-density lipoprotein cholesterol (LDL-C) and confer protection against cardiovascular disease [5]. PCSK9 is secreted by the liver and acts as a natural inhibitor of the LDL receptor (LDLR) pathway, by targeting the receptor to the lysosomal pathway for degradation [3]. Current clinical trials with monoclonal anti-PCSK9 antibodies and SiRNA show that PCSK9 neutralisation is a promising way to achieve low levels of LDL-C in combination with statins [6,7].

Several studies have focussed on the metabolic determinants of plasma PCSK9 concentration. Circulating PCSK9 concentrations are associated with LDL-C, plasma concentrations of triglycerides (TG), glucose and insulin in non-diabetic cohorts [8-11]. The association between plasma PCSK9 and LDL-C, although the most reproducible, is weak as illustrated by the Dallas Heart Study where variations in fasting plasma PCSK9 only accounted for approximately 7% of the variations in LDL-C [11]. At the molecular level, PCSK9 is under the control of the sterol regulatory element binding protein-2 (SREBP-2) [12] pathway and as such it is downregulated by cholesterol [13]. We showed that lipogenic transcription factor SREBP-1c can also regulate PCSK9, acting via the same response element as SREBP-2 in the promoter of PCSK9 [14], suggesting that SREBP-1c might not be dominant under non interventional conditions. One diet that induces SREBP-1 activity is the Fructose enriched diet [15].

We previously showed that hepatic PCSK9 expression is subjected to nutritional regulation, being decreased upon fasting and increased following re-feeding with a high carbohydrate diet in rodents [14]. Insulin increases hepatic PCSK9 expression both *in vitro* in hepatocytes and *in vivo* in mice [14]. Conversely, PCSK9 is repressed by glucagon in rat liver [16]. Accordingly, fasting, but not a ketogenic diet, reduces plasma PCSK9 concentrations in healthy volunteers, with a ≈ 20–35% decrease after 18 h [17,18]. However, so far there are only two reports that describe a dietary modulation of PCSK9 in human. The Mediterranean diet [19] and n-6 PUFAs [20] have been shown to decrease plasma PCSK9 concentrations by ≈ 12% and 13%, respectively.

Here, we assessed the variations of plasma PCSK9 concentrations following various diets that affect differently LDL-C and plasma TG. The aim was to put to test the relationship between PCSK9 and these lipid parameters, as well as the hypothesis that plasma PCSK9 always reflects liver cholesterol synthesis. We used acute fat loads, as well as several short-term dietary interventions (either high-fat or high-fructose diets).

## Methods

### Post-prandial study

Ten healthy volunteers (5 women and 5 men; mean ± SEM age: 25.7 ± 1.5 years) participated in the study approved by the ethical committee of Nantes University Hospital (Protocol referenced as n° 15/06 - BRD 06/3-E). Patients were fasted overnight until 08.00 h, at which time the oral fat load was given. The fat load was 180 g of emulsified blended meal composed of 3.5% dried skimmed milk, 19.25% butter, 23.75% peanut oil, 22% chocolate and 30.25% water. Its energy content was 890 KCal (85% fat, 13% carbohydrates, 2% protein), with 35 g saturated fatty acid, 30 g mono-unsaturated fatty acid, 15 g poly-unsaturated fatty acid and 88 mg cholesterol [21]. Subjects bearing the PCSK9 R104C-V114A double mutant, acting as a dominant negative and severely impairing PCSK9 processing and secretion, were previously described elsewhere [22] . Briefly, subject 1 is a 49 year-old man with low LDL-C (16 mg/dl) presenting with no detectable circulating PCSK9. He has a diabetes mellitus well-controlled with sitagliptine. Subject 2 is his daughter, who has LDL-C values of 44 mg/dl and circulating PCSK9 ~100 ng/ml. Both are heterozygous carriers of a double mutation affecting exon 2 of PCSK9.

### Short-term dietary interventions

Post-hoc measurements of plasma PCSK9 were performed in 2 distinct clinical trials (clinicaltrial.gov identifier NCT00523562 and NCT01119989). Baseline characteristics of the subjects, study design and diet compositions were previously published elsewhere [23-25]. Briefly, 10 healthy male volunteers (age: 24 ± 1 y) took part in the protocol as previously described [23] and received either a hypercaloric high-fat (HF) diet (51.6% saturated fat, 27.6% monounsaturated fat, 8.8% polyunsaturated fat, 376 mg cholesterol), a hypercaloric high-fat/high-protein (HFHP) (52.6% saturated fat, 26.5% monounsaturated fat, 7.9% polyunsaturated fat, 653 mg cholesterol) or an isocaloric control diet (33.5% saturated fat, 37.0% monounsaturated fat, 16.5% polyunsaturated fat, 226 mg cholesterol), for 4 consecutive days (NCT00523562). For the 7-day high fructose diet (HFruc1) (16), 16 healthy male OffT2D (mean ± SEM age: 24.7 ± 1.3 y) and 8 control subjects (mean ± SEM age: 24 ± 1 y) participated in the study and consumed daily, for 7 days, a control diet or an hypercaloric diet enriched with 3.5 g fructose/kg fat free mass (+35% energy intake,) (NCT00523562) [24]. For the 6-day high-fructose diet (HFruc2) [25], 9 healthy male volunteers (age of 23 ± 1 y; BMI: 22.6 ± 0.5 kg/m^2^) were initially included in a randomized, cross-over, single-blinded study. In the present study, dosages of lipids and PCSK9 were performed in only 8 subjects due to an insufficient quantity of serum in samples for one patient. Each participant consumed a control, weight-maintenance diet with a total energy intake corresponding to 1.4 time their resting energy requirements calculated using the Harris and Benedict equation, and containing 55% carbohydrate, 30% lipid, and 15% protein; and a hypercaloric (+36% energy intake) high fructose diet supplemented with 3 g/kg/day fructose and 3 times per day 2.2 g maltodextrin. (NCT01119989). According to a physical examination and a brief medical history, all participants in these 3 studies were in good health and were not taking any medications. The studies were performed on an out-patient basis, and subjects received all their food as pre-packed food items with instructions as how and when to prepare and consume them, and where asked not to consume any other food or drinks. Experimental periods were separated by a washout period of at least 2 weeks (2–10 weeks). All studies were approved by the ethical committee of Lausanne University School of Medicine. All the participants provided written informed consent.

### Analytic procedures

Venous blood samples were obtained after an overnight fast, between 08h00 and 09h00. For plasma PCSK9 dosage, blood was collected in an EDTA tube, maintained at 4°C until plasma and serum were separated and stored at −80°C. Plasma PCSK9 concentrations were assayed in duplicates using a commercially available quantitative sandwich ELISA assay and following the manufacturer instructions (Circulex CY-8079, CycLex Co, Nagano, Japan), as previously described (14). Fasting plasma glucose was determined by the glucose oxidase method (Glucose HK, Roche Diagnostics, Meylan, France). Serum total cholesterol, TG, HDL cholesterol, ApoB and creatinine were measured using routine clinical methods. LDL-C was calculated using the Friedewald equation. Plasma lathosterol extraction and analysis by gas chromatography–mass spectrometry was previously described [26].

### Metabolic investigation

The 2-step hyperinsulinemic euglycemic clamp and ^1^H magnetic resonance spectroscopy (^1^H-MR spectroscopy) used to determine insulin sensitivity, intrahepatocellular lipid (IHCL) and intramyocellular lipid (IMCL) content, were previously published [24]. Briefly, a 2-step hyperinsulinemic euglycemic clamp (0.3 and 1.0 mU.Kg^-1^.min^-1^, 90 minutes each), aimed to achieve glycaemia of 5.5 mmol/l, was performed in combination with measure of hepatic glucose output (6,6 ^2^H_2_ glucose; hot infusion model) [27]. Fasting hepatic insulin sensitivity index was calculated as [100/(hepatic glucose output X insulin)] and whole-body insulin sensitivity from the glucose disposal rate at moderate and high insulinemia. IHCL and IMCL contents were determined by ^1^H-MR spectroscopy on a clinical 1.5 T MR scanner, as described previously [24]. IHCL and IMCL were expressed in units of mmol/kg. Liver spectra were recorded from a large volume (55 cm^3^) during brief respiratory arrests in expiration instead of by a double triggering method. Liver fat content is expressed in units of volume percentage.

### Statistical analysis

All data were expressed as means ± SEMs. The nonparametric Wilcoxon’s signed-paired rank test was used to assess the effect of each dietary intervention. All correlations between plasma PCSK9 and metabolic parameters were assessed using Spearman’s correlation test. A *P-*value ≤ 0.05 was considered statistically significant. Statistical analysis was performed with SAS for Windows version 9.1 Software.

## Results

### Acute oral fat load does not affect plasma PCSK9 concentrations

**Figure 1:**
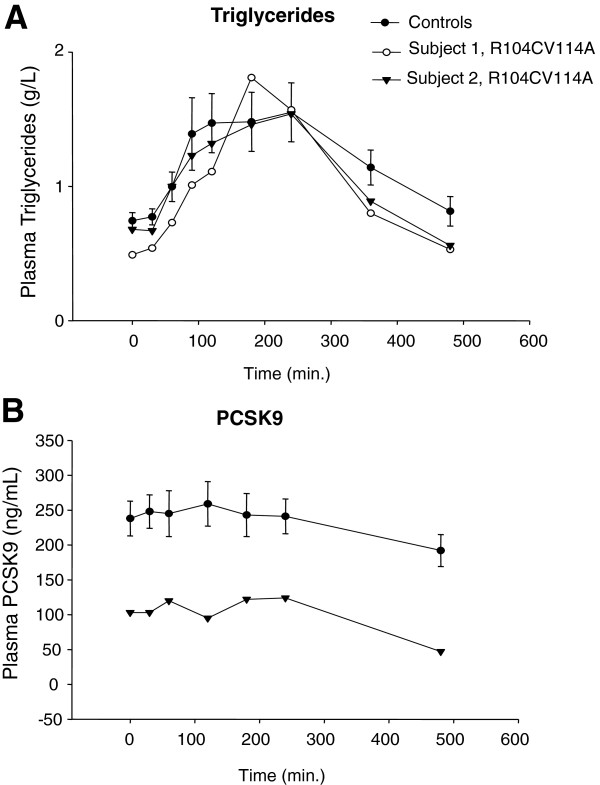
**Oral fat load test in healthy volunteers and two carriers of dominant negative loss-of-function double mutation R104C/V114A.** These subject were previously shown to have no or reduced plasma PCSK9 (22). (**A**) Plasma TG and (**B**) plasma PCSK9 concentrations were determined at various time points after the oral fat load. For the subject 1, plasma PCSK9 was undetectable all through the test. Data represent means ± SEM.

Since we showed that PCSK9-deficient mice have reduced post-prandial hyperlipidaemia following an oral fat load [28], we investigated whether an acute oral fat load can alter PCSK9 plasma concentrations in young healthy volunteers. As expected, after the oral lipid load there was a steep rise in plasma TG concentrations (phase 1) with a peak at 120 min (+ 106% *vs* baseline) followed by a 2h long steady state level (phase 2) and a return to normal levels within 4 h (phase 3) (Figure 1A). Circulating PCSK9 concentrations remained unaltered (Figure 1B) during phase 1 and phase 2, and non-significantly decreased with fasting during phase 3 (−20 % *vs* baseline).

In a step further, we verified whether two subjects bearing the recently described PCSK9 loss of function R104C-V114A double mutant [22] displayed an altered postprandial lipid profile. As showed in Figure 1A, postprandial plasma TG excursion was similar between carriers and non carriers of PCSK9 mutation. As we previously described [22], plasma PCSK9 concentrations remained undetectable in subject 1 and were decreased by ~50% in subject 2 compared to healthy volunteers**.** In subject 2, circulating PCSK9 levels remained stable during phase 1 and phase 2 and decreased by ~50% during phase 3.

### A high fructose diet raises plasma PCSK9 concentrations

**Figure 2:**
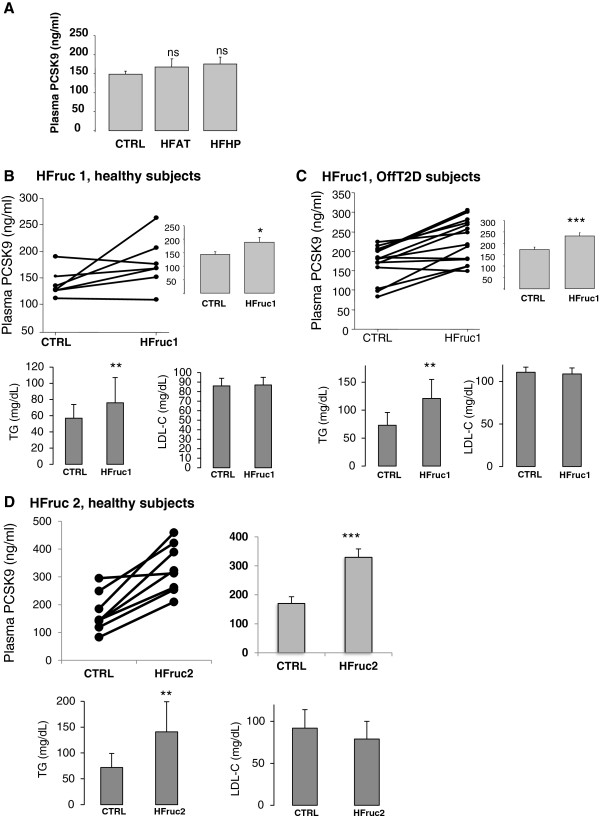
**Effects of short-term dietary interventions.** Fasting plasma PCSK9 concentrations (**A-D**) and LDL-C and plasma TG concentrations (**B-D**) following (**A**) 4-day high fat (HF) or high fat/high protein (HF-HP); 7-day high fructose (HFruc1) diets in either (**B**) healthy volunteers or (**C**) healthy offsprings of type 2 diabetic (OffT2D); (**D**) 6-day high fructose (HFruc2) diet in healthy volunteers. Results are expressed as means ± SEMs. *: p<0.05, **: p<0.01; ***: p<0.001.

We investigated the effect of various short-term diets, including high-fat (HF), high-fat/high-protein (HFHP) and high-fructose (HFruc) diets, on overnight fasted plasma PCSK9 concentrations in young healthy volunteers. Plasma lipid profiles were previously described [23-25] (Figure 2A-D). Briefly, HF diet was associated with increased total-, LDL- and HDL-cholesterol (respectively +9%, p = 0.069, +15%, p = 0.013, +16%, p = 0.006), decreased VLDL-triglycerides (−22%, p = 0.0017) [23]. HFHP diet was associated with increased LDL- and HDL-cholesterol (+20%, p = 0.007, +14%, p = 0.005) [23].

Circulating PCSK9 levels were not significantly altered following both HF and HFHP diets, although there was a trend toward an increase (Figure 2A).

A 7-day hypercaloric high fructose (3.5 g/kg/day) diet (HFruc1) was performed in healthy male volunteers and in healthy OffT2D subjects [24], who are more prone to develop insulin resistance [29-31]. HFruc1 diet promotes a significant rise in plasma TG levels (mean values ± SD in control and HFruc1 diets were respectively: healthy subjects: 57 ± 17 mg/dl and 76 ± 31 mg/dl; p=0.04; OffT2D: 73 ± 23 and 121 ± 34 mg/dl, p<0.001) (Figure 2B-C). HFruc1 diet did not affect LDL-C concentrations (mean values ± SD in control and HFruc1 diets in healthy subjects: 86 ± 8 mg/dl and 87 ± 8 mg/dl, p=ns and in OffT2D: 111 ± 6 and 109 ± 7 mg/dl, p=ns) (Figure 2B-C). PCSK9 levels were significantly increased following HFruc1 diet by 27% in healthy volunteers (mean values ± SD: 139 ± 26 *vs* 177 ± 48 ng/ml, p = 0.05) (Figure 2B) and by 34% in OffT2D subjects (mean values ± SD: 172 ± 44 *vs* 231 ± 54 ng/ml, p = 0.001) (Figure 2C).

In another independent experiment [25], young healthy male volunteers were subjected to a 6-day high fructose diet (3 g/kg/day) (HFruc2). Compared to HFruc1 diet, HFruc2 diet more severely increased plasma TG concentrations (72 ± 27 mg/dl *vs* 141 ± 58 mg/dl, p=0.01), without altering LDL-C levels (mean values ± SD in CTRL and HFruc2 diets were respectively: 92 ± 22 mg/dl and 79 ± 21 mg/dl, p=ns) (Figure 2D). In accordance with HFruc1, fasting plasma PCSK9 concentrations were significantly increased under fructose enriched diet by 93% (mean values ± SD: 170 ± 23 *vs* 329 ± 29 ng/ml, p = 0.004) (Figure 2D).

**Figure 3:**
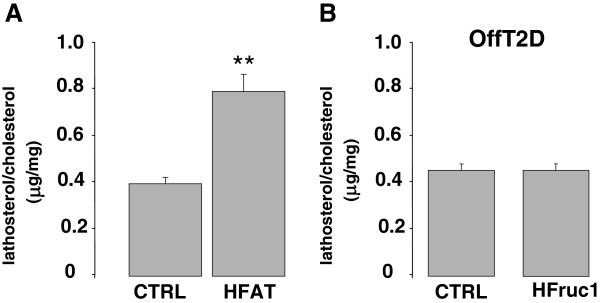
**Effects of short-term high fat (HF) and high fructose (HFRuc1) diets on markers of cholesterol synthesis.** Ratios of lathosterol to cholesterol following (**A**) HF diet in plasma in healthy volunteers and, (**B**) HFruc1 diet in OffT2D subjects. Results are expressed as means ± SEMs. *: p<0.05, **: p<0.01; ***: p<0.001.

Serum ratio to cholesterol of lathosterol is a valid indicator of cholesterol synthesis in human (26). Mean lathosterol to cholesterol ratio was significantly increased by 113% (P=0.008) following the HF diet (Figure 3A), reflecting an increase in cholesterol synthesis. It was not altered upon HFruc1 diet in OffT2D patients (Figure 3B).

### Upon high fructose diet, circulating pcsk9 concentrations are positively associated with hepatic insulin resistance, liver steatosis and vldl-triacylglycerols

**Figure 4:**
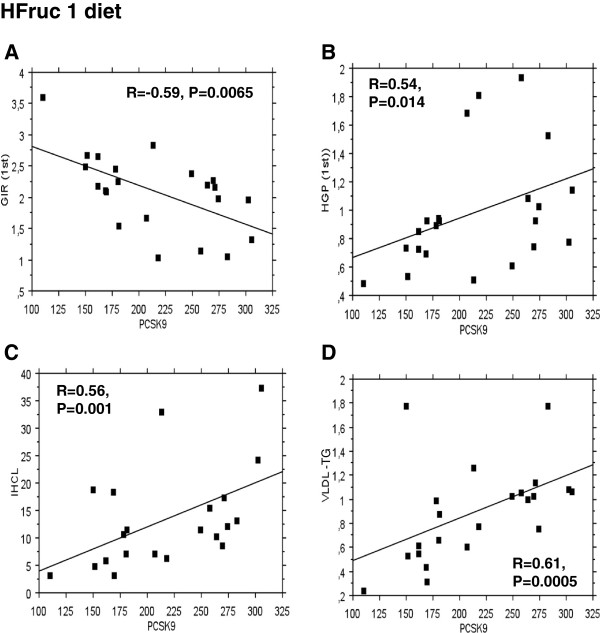
**Correlations between plasma PCSK9 levels (ng/ml) and (A) glucose infusion rate (GIR) during the 1st step of hyperinsulinemic-euglycemic clamp (mg/kg/min), (B) hepatic glucose production (HGP) during the 1st step of hyperinsulinemic-eugyclemic clamp (mg/kg/min), (C) intrahepatocellular lipid content (IHCL)(mmol/kg), and (D) VLDL-TG (mmol/l) in healthy volunteers (n=7) and OffT2D subjects (n=15) under a HFruc diet.** Correlations were made using Spearman’s correlation test.

Recent data in large cohorts have showed that plasma PCSK9 concentrations are positively correlated with the homeostasis model assessment-insulin resistance (HOMA-IR) index, which is an indirect marker of insulin sensitivity [9-11]. Here, we performed a 2-steps hyperinsulinemic-euglycemic clamp, which is the gold standard to assess the insulin sensitivity [32]. In healthy volunteers (n=22), with (OffT2D: n=15) and without (n=7) a family history of type 2 diabetes mellitus, fed a control diet, there was no association between circulating PCSK9 concentrations and both whole-body (i.e., GIR: glucose infusion rate) and hepatic (HGP: hepatic glucose production) insulin sensitivity (Table 1). Moreover, PCSK9 concentrations were not correlated with the ectopic lipid deposition in liver (IHCL) and skeletal muscle (IMCL), as well as with plasma lipid parameters (Table 1). Upon HFruc1 diet there was a significant association between overnight-fasted plasma PCSK9 concentrations and both GIR and HGP under low-insulin infusion dose during the clamp (Table 1 and Figure 4A-B). In addition, circulating PCSK9 was positively correlated with IHCL and with fasting VLDL-TG concentrations (Table 1 and Figure 4C-D). There was no statistically significant association between PCSK9 and LDL-C or HDL-C under the same conditions (Table 1).

**Table 1:** Correlations between plasma PCSK9 and metabolic parameters

**correlation with PCSK9**	**CTRL**			**HFruc1**		
	**Rho de Spearman**	**p-value**	**n**	**Rho de Spearman**	**p-value**	**n**
FPG (mg/dl)	−0,040	0,859	22	0,266	0,232	22
GIR (1st)	−0,084	0,710	22	−0,593	**0,004**	22
GIR (2nd)	0,045	0,844	22	−0,439	**0,041**	22
HGP	−0,189	0,412	22	−0,019	0,934	22
HGP (1st)	0,003	0,991	22	0,542	**0,009**	22
HGP (2nd)	−0,143	0,526	22	0,116	0,608	22
FFA (mmol/l)	−0,317	0,150	22	−0,054	0,811	22
IHCL	0,182	0,417	22	0,558	**0,007**	22
IMCL	0,381	0,080	22	0,195	0,385	22
CT(mg/dl)	0,095	0,673	22	0,364	0,096	22
LDL-C (mg/dl)	0,178	0,427	22	0,344	0,117	22
TG (mg/dl)	0,267	0,230	22	0,538	**0,010**	22
VLDL-TG (mg/dl)	0,341	0,120	22	0,608	**0,003**	22
HDL-C (mg/dl)	−0,343	0,118	22	0,006	0,980	22

Spearman’s correlations between plasma PCSK9 levels and metabolic parameters in healthy volunteers (n=7) and offsprings of type 2 diabetic patients (n=15) after an isocaloric control (CTRL) or a high fructose (HFruc1) diet [24]. Data from both groups of patients were pooled to increase statistical power. GIR: glucose infusion rate, HGP: hepatic glucose production, IHCL: intrahepatocellular lipid content, IMCL: intramyocellular lipid content, FPG: fasting plasma glucose, 1^st^ indicates the first step (0.3 mU.kg^-1^.min^-1^) and 2^st^ the second step (1.0 mU.kg^-1^.min^-1^) of the hyperinsulinemic-euglycemic clamp.

## Discussion

Recent studies have focused on the association between circulating PCSK9 concentrations and metabolic parameters in human [8-11]. Plasma PCSK9 has been found to be consistently associated with LDL-C, and less robustly with TG, fasting plasma glucose and HOMA-IR. The major finding of our studies is that plasma concentrations of PCSK9 were induced in response to short-term HFruc diets by 27 to 93% in healthy volunteers (Figure 2B-D). Circulating PCSK9 levels were associated with both whole-body, hepatic insulin resistance, liver steatosis and VLDL-TG (Figure 4 and Table 1). In accordance with a regulation of PCSK9 by carbohydrate intake, we previously demonstrated that high-carbohydrate refeeding in mice [14] increases hepatic PCSK9 mRNA and protein levels. Our study does not explain whether changes in plasma PCSK9 upon a high-fructose diet are causative of the variations in VLDL-TG and what could be the molecular mechanisms involved, in particular whether PCSK9 acts upon hepatic VLDL production. In humans, using lipoprotein kinetics with stable isotopes, we observed an increase of VLDL production in 2 family members with PCSK9 GOF variant S127R [33] but it is unclear whether this is related to this specific variant or to a general trait of PCSK9 GOF variants. In mice, we showed that PCSK9 overexpression is accompanied with hypertriglyceridemia due to VLDL overproduction. However this phenotype was restricted to fasted mice, and was not observed in fed mice [34]. Interestingly, we showed that PCSK9 is normally decreased during fasting [14]. We hypothesized that VLDL production was increased due to a lack of re-uptake of nascent VLDL by the LDLR (as described by Twisk J. et al. [35]). Indeed, fasting seemed to increase the effect of PCSK9 on the LDLR degradation and these mice had virtually no LDLR in their liver compared with fed mice that overexpressed PCSK9. Fructose inhibits hepatic lipid oxidation and favors VLDL-TG-synthesis [36] and it cannot be excluded that PCSK9 was associated with nascent VLDL particles produced by the liver. However, whether PCSK9 is physically associated with lipoproteins remains a controversial issue [8,37].

Plasma PCSK9 follows a diurnal rhythm that parallels fluctuations of lathosterol to cholesterol ratio [18]. Cholesterol synthesis is driven by SREBP-2 that translocates to the nucleus in response to lower cholesterol content of the endoplasmic reticulum membrane and activates HMG-Coa reductase. SREBP2 also up-regulates PCSK9 [12,39] and LDLR expression [39]. In order to estimate how the present diets affect cholesterol synthesis, we measured serum ratios of lathosterol to cholesterol as a surrogate marker of cholesterol synthesis [40]. There was an increase upon HF diets, as previously described [41] (Figure 3A). This might relate to the non-significant trend towards an increase of plasma PCSK9 we observed. It is possible that the trend would turn out to be significant with more subjects. However, upon HFruc diet, there was no change in cholesterol synthesis (Figure 3B), but plasma PCSK9 increased significantly, suggesting that SREBP-2 pathway is not responsible for these changes. We showed that SREBP-1c is able to drive the expression of PCSK9 and that SREBP2 and SREBP1c share the same response element on the promoter of PCSK9 [14,42]. Recent studies in hamsters also support the implication of SREBP-1c in PCSK9 regulation [43]. Diurnal fluctuations of PCSK9 that parallel cholesterol synthesis suggest that SREBP2 pathway is dominant over SREBP-1c activation under non-interventional conditions. Our finding that plasma PCSK9 is increased by a high fructose diet but that cholesterol synthesis is not affected (Figure 3B) suggests that PCSK9 regulation is not dependent upon SREBP-2 under this specific diet. Because SREBP-1c is induced by a high fructose diet [15], it might be responsible for the increase of plasma PCSK9.

Our study underlines the disconnection that might take place between PCSK9 and LDL-C level under specific nutritional conditions. Indeed, the increase of LDL-C (reported in [23]) under a HF diet was not linked to an increase of PCSK9. Conversely, the increase of PCSK9 under HFruc diet was not associated to an increase of LDL-C (Figure 2B-D). It is surprising that the large increase of circulating PCSK9 seen under fructose (up to 93% in healthy volunteers in HFruc2) was not associated to an increase of LDL-C (Figure 2D). Further studies are needed to unravel the molecular mechanisms involved in this disconnection. It is also unclear why the two studies led to such different magnitude of increase in PCSK9 (23% for the 7 day-long *vs* 93% for the 6 day-long diet). Subjects had on average similar basal concentrations of PCSK9. It is possible that a peak of concentration occurs at day 6 or before. However, HFruc2 induced a higher hypertriglyceridemia than HFruc1 (+33% in HFruc1 Healthy Patients *vs* +107% in HFruc2 [25]), suggesting a better efficacy of the diet.

Several elements suggested a potential association between PCSK9 and postprandial lipidaemia, in majority represented by chylomicrons and their remnants. First PCSK9 might influence chylomicron clearance by degrading the LDLR, although there is conflicting data in Familial Hypercholesterolemia patients on the role of the LDLR in chylomicrons clearance [44,45]. Second, we showed that *PCSK9*-deficiency is associated with reduced postprandial hyperlipidaemia in mice challenged with an olive oil bolus, due to decreased apoB output and a modification of chylomicron size, number and catabolism [28]. Here, we failed to detect any variation in plasma PCSK9 concentrations following the acute oral fat load in healthy volunteers (Figure 1B). In addition, we found that 2 subjects with *PCSK9* LOF mutation responded in a similar fashion than controls. It is possible that an olive oil load, similar to what we did in PCSK9 knockout mice [28], would have changed the outcome of the investigation in these 2 subjects. However, these subjects cannot be considered as entirely deficient for PCSK9 because it is unclear how much wild type PCSK9 is present in the cells of these individuals and because some wild type protein is still being secreted for one of them. The R104CV114A variant is not cleaved and not secreted. The variant exerts a dominant negative effect over the wild type protein [22]. Carriers of PCSK9 R104CV114A have different concentrations of plasma PCSK9 despite being both heterozygous for the mono-allelic double mutation. For one of them PCSK9 was virtually absent from the blood, while for the other carrier concentrations were around 100 ng/ml. We hypothesized that this variability is due to the dominant effect of the variant [22]. Because the variant is not secreted, we assume that plasma PCSK9 in these subjects is the wild type protein. If plasma PCSK9 had a role in postprandial lipemia, these two subjects would have had a different response to the oral fat load. Of course, some limitations to our study are to be taken into account, as discussed below. All together, our data suggest that plasma PCSK9 is not associated to postprandial hyperlipidaemia in human.

Recent studies suggest that PCSK9 may interfere with glucose homeostasis, since: i) insulin increases PCSK9 expression *in vitro* in hepatocytes and *in vivo* in mice and rats [14,16]; ii) the expression of PCSK9 is altered in rodent models of diabetes [46]; and iii) circulating PCSK9 concentrations were found to be correlated with the level of insulin sensitivity assessed by the HOMA-R index both in adults [11] and in children and adolescents [9]. A recent phenotyping of *PCSK9*-deficient mice revealed that they were hypoinsulinemic, hyperglycaemic and glucose intolerant [47]. Our own investigations in mice with a different genetic background didn’t point out any obvious abnormality in terms of glucose homeostasis and pancreatic beta cell function [48]. High fructose intake leads to hypertriglyceridemia and hepatic insulin resistance and obesity [49]. Whether the molecule of fructose itself is responsible for these deleterious effects is not established because high sucrose diets leads to similar defects [49].

We show here that PCSK9 was only positively associated with both whole-body and hepatic insulin resistance in healthy volunteers (including OffT2D) when they fed a short-term HFruc diet, but not under basal conditions (Figure 4 and Table 1). Additionally, we found that PCSK9 is associated with liver steatosis upon HFruc diet, without any correlation with IMCL. Previous characterizations of these subjects [23,24] showed that hepatic steatosis was not accompanied with hepatic insulin resistance when induced by the HF, HFHP diets but that it was under the HFruc diet. Such a positive association between circulating PCSK9 levels and liver TG content assessed by proton magnetic spectroscopy was previously described in the cohort of the Dallas Heart Study, although the level of the correlation coefficient was weak (r = 0.13) [11]. In accordance with a potential link between PCSK9 and liver steatosis, we recently described a positive association between PCSK9 and gamma-glutamyl transferase levels, a marker of hepatic steatosis, in type 2 diabetic patients [50].

Finally, our study had certain limitations. Although our metabolic phenotyping was exhaustive, the number of subjects is small and potentially limited the ability to detect weak correlations. In addition, the duration of each diet is short (≤ 7 days) and additional studies with extended periods of dietary intervention need to be performed to confirm our observations. The variation in the extent of fructose-induced PCSK9 expression between the HFruc1 (+23%) and HFruc2 (+93%) diets is surprising since both diets were similar in term of fructose and energy intake. The only difference was the addition of maltodextrin in HFruc2 diet. In parallel with a higher increase of PCSK9, the hypertriglyceridemia was also more robust in HFruc2 diet. While some of our preliminary observations in a small number of healthy volunteers (n=6) suggest that high glucose diet also increase circulating PCSK9 levels (+47%, p=0.17) (*data not shown*), it would be interesting to confirm this observation in a larger number of subjects. Although our findings suggest that plasma PCSK9 concentrations do not parallel cholesterol synthesis under a high fructose diet, these studies were not designed to explore these aspects. In particular diurnal rhythms of cholesterol synthesis and plasma PCSK9 concentrations were not determined in these patients. Concerning the effect of *PCSK9*-deficiency on post-prandial lipid profile, we only investigated two subjects from the same family with the same *PCSK9* LOF and that were not from the same gender. It cannot be excluded that different conclusions would emerge from a study with more subjects or with subjects with a different LOF mutation.

In summary, we demonstrated that circulating PCSK9 levels are significantly increased following a short-term high-fructose diet. Under these specific nutritional conditions, PCSK9 concentrations were positively correlated with insulin resistance, liver steatosis and VLDL-TG concentrations.

## Competing interest

BC has served on the advisory panel for Sanofi-Regeneron and Amgen. Kim-Anne Lê is presently an employee of Nestec Ltd. The others authors declare that they have no competing interests.

## Authors’ contributions

The author’s responsibilities were as follows : BC and PC were responsible for the design of the study, analyzed the results and prepared an initial draft of the manuscript which was reviewed and modified by all authors; CL, MLB and CLM performed the PCSK9 dosage; YZ, BC and MK recruited the subjects and performed the acute oral lipid load; MB, KAL, FT and LT designed and performed the clinical trial with the HF, HFHP and HFruc diets; CB and RK measured IHCL and IMCL by ^1^H-MRS and analyzed the data, BGD was responsible for the statistical analysis. All authors read and approved the final manuscript.
